# Stoking an anti-liver cancer immune response with cryoablation plus an intratumoral TLR9 agonist and dual checkpoint inhibitors

**DOI:** 10.1007/s00262-025-04286-8

**Published:** 2026-02-12

**Authors:** Yunpeng Yang, Tyler Mandt, David Mittelstein, Manasi Das, Panyisha Wu, Mansur A. Ghani, Ritvik Illindala, Nicole F. Steinmetz, Adam M. Burgoyne, Zachary Berman, Nicholas Webster, Isabel G. Newton

**Affiliations:** 1 Department of Radiology, Division of Interventional Radiology, University of California San Diego, San Diego, CA USA; 2 VA San Diego Healthcare System, 3350 La Jolla Village Drive, San Diego, CA 92161 USA; 3 Department of Medicine, University of California San Diego, La Jolla, CA USA; 4 Research Service, Jennifer Moreno VA Medical Center, San Diego, CA USA; 5 Moores Cancer Center, University of California San Diego, La Jolla, CA USA; 6 Division of Hematology/Oncology, Department of Medicine, University of California San Diego, La Jolla, CA USA; 7 School of Medicine, University of California San Diego, La Jolla, CA USA; 8 Aiiso Yufeng Li Family Department of Chemical and Nano Engineering, University of California San Diego, La Jolla, CA USA; 9 Department of Bioengineering, University of California San Diego, La Jolla, CA USA; 10 Center for Nano-ImmunoEngineering, University of California San Diego, La Jolla, CA USA; 11 Center for Engineering in Cancer, Institute for Engineering in Medicine, University of California San Diego, La Jolla, CA USA; 12 Shu and K.C. Chien and Peter Farrell Collaboratory, University of California San Diego, La Jolla, CA USA; 13 Institute for Materials Discovery and Design, University of California San Diego, La Jolla, CA USA

**Keywords:** Hepatocellular carcinoma, Cryotherapy, CpG, Dual checkpoint blockade, Abscopal

## Abstract

The rising incidence of hepatocellular carcinoma (HCC) is partly driven by metabolic dysfunction-associated steatohepatitis (MASH). Recurrence after treatment is high, and advanced disease carries a poor prognosis. The immunosuppressive tumor microenvironment (TME) in HCC limits the efficacy of immunotherapy, necessitating innovative approaches. To evaluate the efficacy of a novel therapeutic strategy designed to stimulate an antitumor immune response by combining cryoablation (Cryo) to release tumor antigen with an intratumoral immunostimulant (CpG) to facilitate immune recognition and dual immune checkpoint inhibitors (CPI) to disinhibit T cells. RIL-175 cells were orthotopically injected into two liver lobes in mice on a MASH-inducing diet. One tumor in each mouse was treated with partial Cryo, systemic dual CPI (anti-PD-1 and anti-CTLA-4), intratumoral TLR9 agonist CpG, or combinations thereof, while the second tumor was untreated. Tumor growth was monitored prior to and after ablation. Immune cell profiles and cytokine levels in both treated and untreated tumors were analyzed postmortem. The combined treatment of Cryo with CpG and dual CPI achieved the strongest tumor control and survival benefit. Cryo alone increased growth of the untreated tumor but addition of CpG and CPI reversed this effect. Immunologically, CPI primarily drove CTL expansion and PD-1 modulation, whereas CpG suppressed Tregs. Cryo monotherapy leads to immunosuppression, characterized by elevated Tregs, MDSCs, and PD-1 expression. Combining Cryo with CpG and CPI counteracts the inherent limitations of each therapy and enhances systemic anti-tumor immune responses.

**Supplementary Information:**

The online version contains supplementary material available at 10.1007/s00262-025-04286-8.

## Introduction

Hepatocellular carcinoma (HCC) poses a growing healthcare burden in the United States, with rising incidence and mortality [1]. Despite curative-intent treatments such as surgical resection or tumor ablation, recurrence occurs in up to 60–85% of cases [2–4]. In rare instances, ablation can trigger an “abscopal effect”—a systemic immune response resulting in regression of distant metastases [5]. However, incomplete ablation can stimulate tumor proliferation [6]. Cryoablation (Cryo) is a promising therapy as it destroys tumors through lethal freezing, releasing intact tumor-associated antigens (TAAs) and initiating a local inflammatory response involving IL-1, IL-6, NFκB, and TNF-α [7]. Antigen-presenting cells (APCs) then process TAAs and present them to T cells in lymph nodes as a step towards priming anti-tumor immunity.

The immunosuppressive liver microenvironment and HCC’s capacity for immune evasion, however, contribute to high recurrence rates [8]. Regulatory T cells (Tregs) and myeloid-derived suppressor cells (MDSCs) dampen immune activation, while PD-L1 expression on tumor cells binds PD-1 on cytotoxic T cells, promoting T cell exhaustion [9]. Similarly, CTLA-4 impairs T cell priming by blocking CD28 binding to costimulatory molecules on APCs. Immune checkpoint inhibitors (CPI) targeting PD-1 can potentially reverse T cell exhaustion, while CTLA-4 blockade enhances T cell priming and suppresses Tregs [10]. These immunotherapies have had limited success in HCC.

While single-agent CPIs yield modest responses in HCC, combination therapies have shown greater efficacy. Dual blockade of PD-L1 and VEGF [11] or CTLA-4 [12] has produced improved outcomes. The landmark HIMALAYA trial evaluated the STRIDE regimen—single-dose tremelimumab (anti-CTLA-4) combined with durvalumab (anti-PD-L1) every four weeks—and demonstrated superior overall survival versus sorafenib (16.4 months vs 13.8 months) in patients with unresectable HCC [13]. Thus, the FDA approved STRIDE as first-line therapy for unresectable HCC. In CheckMate-040 trial [14], nivolumab plus ipilimumab showed ORRs of ~ 27–32% across arms, whereas prior data of nivolumab monotherapy in sorafenib-treated HCC reported ORRs around ~ 14%. Similarly, in a phase I/II study [12], the combination of tremelimumab and durvalumab demonstrated superior antitumor activity compared with either agent alone in patients with unresectable hepatocellular carcinoma. Collectively, these pivotal studies support the enhanced efficacy of dual checkpoint blockade over monotherapy in advanced HCC and provide the rationale for selecting dual CPI as the immunotherapy backbone in our study.

Alternatively, the tumor microenvironment (TME) can be remodeled with CpG oligodeoxynucleotides (CpG-ODN), agonists of Toll-like receptor 9 (TLR9), that activate plasmacytoid dendritic cells (pDCs) and enhance TAA presentation while promoting proinflammatory cytokine production (signaling through type I interferons) [15, 16]. Though shown to be safe in early clinical trials, CpG monotherapy has limited efficacy [17]. Its effectiveness depends on the timing of TAA release and the immune activation state [18]. In metastatic melanoma, CpG has demonstrated synergy with CPI [19, 20].

We hypothesize that cryoablation releases tumor antigens, intratumoral CpG enhances local antigen presentation and dendritic cell activation, and dual checkpoint blockade restores effector T-cell function. Together, these additive effects may overcome immune suppression and elicit a robust antitumor response in a murine model of multifocal HCC.

## Materials and methods

### Study design and orthotopic HCC model

All animal procedures were approved by the UCSD Institutional Animal Care and Use Committee (IACUC) under protocol number S06319. All procedures were performed in accordance with institutional guidelines and adhered to the ARRIVE 2.0 guidelines. This study utilized 104 male C57BL/6 J mice (Jackson Laboratory). Female mice were excluded due to the poor engraftment rates of RIL-175 cells, which originated in a male mouse. At 8 weeks of age, the mice were placed on a western diet (D12079, Research Diets, New Brunswick, New Jersey) to induce metabolic dysfunction-associated steatohepatitis (MASH). After 4 weeks mice were implanted with RIL-175 cells [21] in two separate lobes of the liver by ultrasound-guided orthotopic injection.

**Fig. 1:**
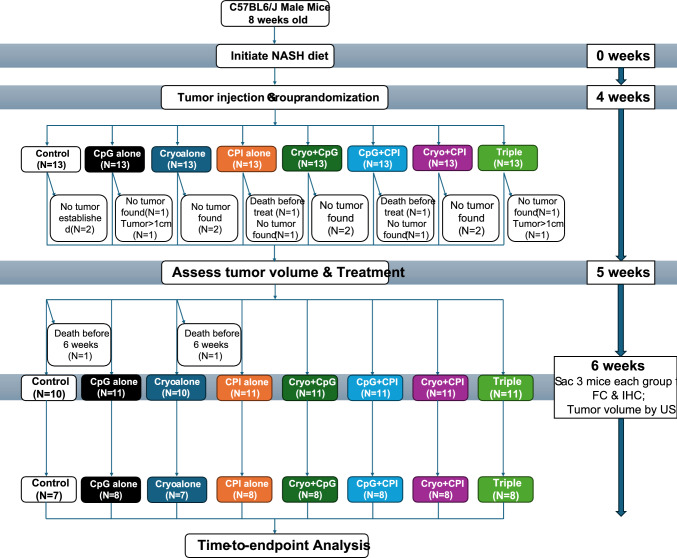
Experimental design and treatment timeline of the orthotopic HCC mouse model. One Control and one Cryo mouse died before the 6-week endpoint; both were included in the time-to-endpoint analysis but excluded from relative tumor volume assessments. (FC: flow cytometry; IHC: immunohistochemistry stain; US: ultrasound)

Mice were randomized into 8 groups (Fig. 1):

- *Control (Ctrl)* Receive mock CpG and mock CPI treatments plus a sham procedure.
- *CpG only* Receive CpG and mock CPI and a sham procedure.
- *Cryo only* Undergo Cryo with mock CpG and mock CPI.
- *CPI only* Receive CPI plus mock CpG and a sham procedure.
- *Cryo + CpG* Undergo Cryo and CpG with mock CPI.
- *Cryo + CPI* Undergo Cryo and CPI with mock CpG.
- *CpG + CPI* Receive CpG and CPI and a sham procedure.
- *Cryo + CpG + CPI (Triple therapy)* Cryo plus intratumoral CpG and systemic CPI.

The three treatments were as follows:

- *CpG* 100 μg of CpG-ODN injected in 3 fractions along the tumor ablation margins and in the tumor under US guidance. For the mock CpG, the same volume of saline was delivered under identical conditions.
- *CPI* 125 µg of anti-mouse PD-1 antibody and 250 µg of anti-mouse CTLA-4 (CD152) antibody were delivered by intraperitoneal injection on Day 0 and anti-PD-1 antibody was re-administered every 3 days for 2 weeks total. For the mock CPI, an equal volume of 0.9% endotoxin-free saline was delivered under identical conditions. The dosages and schedule were selected based on preliminary dose-finding experiments and previously published studies employing anti-PD-1 and anti-CTLA-4 antibodies in murine tumor models [22–24], with minor modifications to optimize treatment efficacy in our model.
- *Cryo* the Galil cryoablation system (Boston Scientific) with a 17G, 3-cm IceSeed probe was used to perform three cycles of one-minute freezes at 50% power to achieve incomplete (75% or less) tumor ablation. For the sham Cryo, the cryoprobe was inserted for the same duration but without activating it.

Periprocedural Care and Anesthesia.

All procedures were performed under general anesthesia with isoflurane (3% induction, 1.5% maintenance in 2 L/min O₂ via nasal cone) and supplemented with local anesthesia using 1% lidocaine at the cryoablation site [25, 26]. Analgesics were administered prior to cryoablation to minimize procedural pain. Body temperature was maintained with Deltaphase heating pads, and the depth of anesthesia was monitored throughout by assessing movement response to tail pinch and respiratory rate. All procedures were conducted using aseptic technique and in accordance with institutional animal care standards to ensure welfare and procedural consistency.

### Tumor volume characterization and endpoints

Tumor volumes were assessed by ultrasound, with the largest axial and sagittal dimensions recorded. Only tumors ≥ 5 mm were eligible for treatment. At treatment initiation, tumor sizes were comparable across groups. To adjust for baseline variability, fold changes were calculated by comparing tumor volumes one-week post-treatment to baseline. Mice were sacrificed if tumors exceeded 1 cm in any dimension or if they showed signs of distress, including impaired mobility, poor grooming, weight loss, or visible injuries. Study design details, including group allocation and exclusion criteria, are shown in Fig. 1.

### Flow cytometry and histology

Seven days after treatment initiation, 3 mice per group were euthanized under isoflurane anesthesia followed by cervical dislocation for flow cytometry and histology. Tumors were bisected; one half was fixed in 4% formaldehyde, paraffin-embedded, sectioned, and stained with H&E and anti-CD8 IHC (Cat# ab183685, Abcam). The other half and peripheral blood were processed for flow cytometry. Tumors were minced and digested in 1% collagenase (37 °C, 30 min), filtered through a 100-μm strainer (Thermo Fisher), and RBCs lysed with ACK buffer. Immune cells were enriched via 40%-70% Percoll gradient centrifugation (1000 × g, 30 min, brake off) and collected from the interface. Cells were stained using two panels: T cell (including peripheral blood) and myeloid cell. The T cell panel included antibodies against CD4 (clone RM4-5, 100,548), CD8a (53–6.7, 100,750), CD25 (PC61, 102,026), CD44 (IM7, 103,056), CD62L (MEL-14, 104,410), CD69 (H1.2F3, 104,545), CD137 (17B5, 106,105), PD-1/CD279 (29F.1A12, 135,213), CTLA-4/CD152 (UC10-4B9, 106,314), FoxP3 (MF-14, 126,408), and CD45 (30-F11, 103,151). The myeloid panel included antibodies against CD11b (M1/70, 101,236), F4/80 (BM8, 123,110), Ly6G (1A8, 127,614), Ly6C (HK1.4, 128,041), Gr1 (RB6-8C5, 108,442), and CD45 (30-F11, 103,132). All antibodies and viability dye were purchased from BioLegend (San Diego, CA). Flow cytometry data were acquired on a CytoFLEX flow cytometer (Beckman Coulter) and analyzed using FlowJo software (Ashland, Oregon).

### Chemokine/Cytokine analysis

Chemokine and cytokine levels were quantified using an electrochemiluminescence-based multiplex assay (MESO QuickPlex SQ 120MM; Meso Scale Diagnostics). Key analytes including GM-CSF, IL-2, IL-6, IL-10, CXCL1, TNF-α, IL-12p70, IL-13, and IL-4 were measured using the U-PLEX Custom Biomarker Group 1 Assay (Cat# K15069M-1). All procedures followed the manufacturer's protocols.

### Data analysis and statistical methods

Statistical analyses were performed using Prism 10 (GraphPad, San Diego, CA). One-way ANOVA with Tukey’s post hoc test was used to analyze tumor volume changes and flow cytometry data across treatment groups, separately for treated and satellite tumors. Two-way ANOVA with Šídák’s correction was used to compare treated vs. satellite tumors within each group. Three-way ANOVA assessed main and interaction effects of Cryo, CpG, and CPI on tumor growth and immune parameters, with Tukey’s test for post hoc comparisons. Survival was analyzed using the Kaplan–Meier method and compared by log-rank (Mantel–Cox) test.

Correlation analyses were performed to assess the relationships between immune parameters in peripheral blood and tumors, as well as between systemic cytokines and intratumoral immune features. Spearman’s rank correlation (ρ) was used for nonparametric associations, with *P* values and 95% confidence intervals reported. Nonlinear regression was applied where appropriate, with model fit assessed by the coefficient of determination (R^2^).

A full description of the methods can be found in the Supplementary.

## Results

### Combination of Cryo TLR9 immunostimulation with CpG, and dual checkpoint inhibition (CPI) led to maximum tumor growth abrogation

**Fig. 2:**
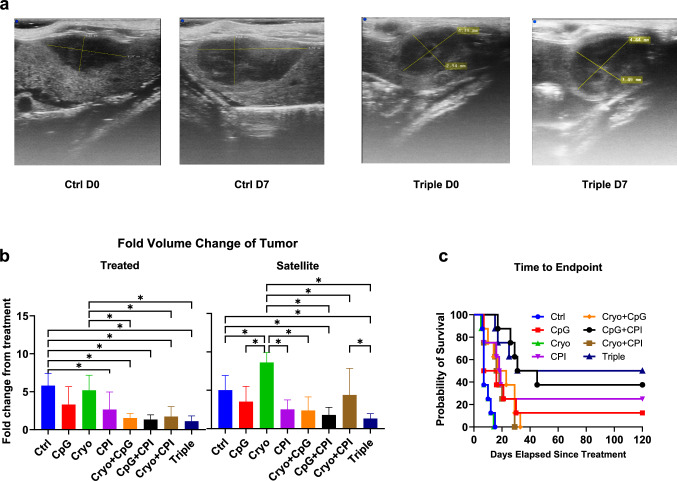
Cryoablation combined with CpG and dual checkpoint inhibition suppresses tumor growth and prolongs survival. **a** Representative ultrasound images of satellite tumors from Control and Triple therapy groups at Day 0 and Day 7. **b** Fold-change in tumor volume one week post-treatment for treated and satellite tumors across 8 groups. Cryo increased satellite tumor growth 1.7-fold vs. Control (*P* = 0.014), significantly more than Cryo + CPI (1.96-fold), CPI (3.4-fold), Cryo + CpG (3.6-fold), CpG + CPI (4.8-fold), and Triple (6.6-fold) (all *P* ≤ 0.001). **c** Kaplan–Meier survival curves by treatment group. (*P < 0.05)

To evaluate treatment effects on tumor growth, fold-volume changes were measured 1-week post-treatment (Fig. 2a). CPI alone or in combination significantly reduced treated tumor growth versus Control: 2.18-fold (CPI, *P* = 0.007), 3.4-fold (Cryo + CPI), 3.8-fold (Cryo + CpG), 4.3-fold (CpG + CPI), and 5.2-fold (Cryo + CpG + CPI) (all *P* < 0.001; Fig. 2b). No significant differences were observed among CPI-containing groups. Cryo or CpG alone had no effect.

Cryo increased satellite tumor growth 1.7-fold over Control (*P* = 0.014). Adding CpG or CPI prevented this effect, while combining all three reduced satellite tumor growth by 2.8-fold compared to Control (*P* = 0.028).

A three-way ANOVA identified main effects of CPI and CpG in both treated and satellite tumors (Supplementary Fig. 1a, 1b) with a weaker effect of Cryo.

Time-to-endpoint was assessed by Kaplan–Meier analysis (Fig. 2c). Compared to Control, time-to-endpoint was prolonged in the CpG, CPI, CpG + CPI, and Cryo + CpG + CPI groups, with hazard ratios of 0.464 (*P* = 0.04), 0.303 (*P* = 0.003), 0.184 (*P* < 0.001), and 0.171 (*P* < 0.001), respectively. Median survival was 7 days in the Control, CpG, and Cryo groups, and was extended to 17–29 days with CPI-containing treatments. Notably, 50% of mice in the Triple therapy (Cryo + CpG + CPI) group survived beyond 120 days.

### CPI increases CTLs and Cryo + CpG promotes early CTL activation in treated tumors

**Fig. 3:**
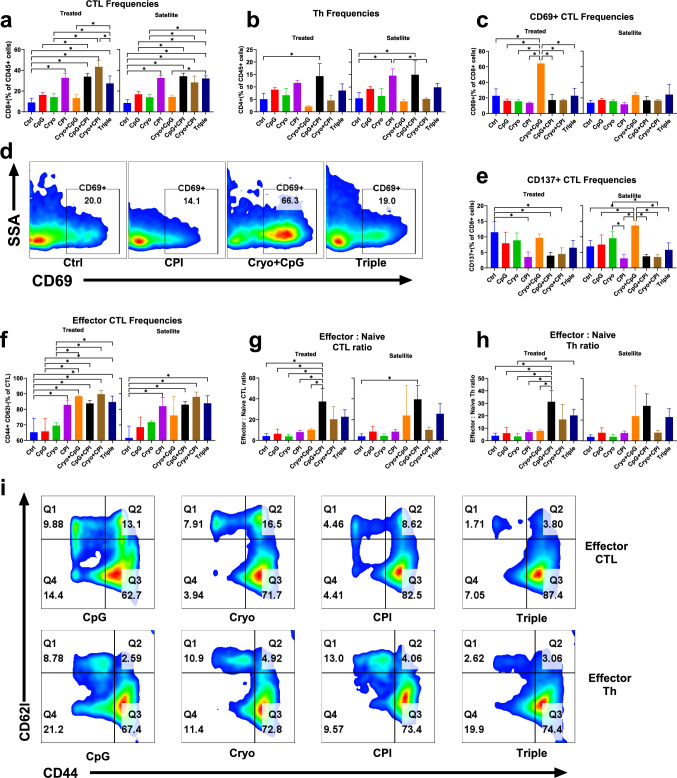
Combination treatments enhance intratumoral T cell activation and effector differentiation. **a**, **b** Intratumoral CTL (CD8^+^, **a**) and Th (CD4^+^, **b**) frequencies as a percentage of CD45^+^ cells across groups. **d** Representative flow cytometry plots of CD69 expression on CD8^+^ T cells from treated tumors in the Control, CPI, Cryo + CpG, and Triple groups. **c**, **e** Early activated CD69^+^ **c** and fully activated CD137^+^ **e** CTLs analyzed across groups.** f** Effector CTL frequencies analyzed across groups. All CPI-containing treatments (CPI, Cryo + CPI, CpG + CPI, Triple) significantly increased effector CTLs compared to Control in both treated (mean differences: 17.6%, *P* = 0.007; 24.4%, *P* = 0.003; 18.47%, *P* = 0.005; 19.4%, *P* = 0.003) and satellite tumors (20.37%, *P* = 0.02; 26.4%, *P* = 0.002; 21.37%, *P* = 0.014; 22.3%, *P* = 0.01). **g**, **h** Effector-to-naïve ratios for CTL(g) and Th(h) cells across groups.** i** Representative CD44/CD62L flow cytometry plots for selected groups (CpG, Cryo, CPI, Triple). The upper row shows CD8^+^ cytotoxic T lymphocytes (CTLs), and the lower row shows CD4^+^ helper T cells (Th). Naïve T cells (CD44⁻CD62L^+^) are located in the upper left (Q1) quadrant, central memory T cells (CD44^+^CD62L^+^) in the upper right (Q2) quadrant, and effector T cells (CD44^+^CD62L⁻) in the lower right (Q3) quadrant. (*P < 0.05)

Cytotoxic T-cell lymphocyte (CTL) frequencies were analyzed by flow cytometry. All CPI-treated groups (CPI, Cryo + CPI, CpG + CPI, Cryo + CpG + CPI) showed increased CTLs versus Control in both treated and satellite tumors, while CpG alone had no effect (Fig. 3a). Three-way ANOVA identified CPI as the dominant factor, accounting for 77.2% and 85.5% of the variance in treated and satellite tumors, respectively (*P* < 0.001; Supplementary Fig. 2a, b). Pooled analysis confirmed CPI-driven increases (21.2% and 18.4%; both *P* < 0.001; Supplementary Fig. 3b).

Early CTL activation was assessed by CD69 expression. Cryo + CpG induced the highest early CTL frequencies in treated tumors (Fig. 3c, d), an effect that was reduced with the addition of CPI as Triple therapy (− 41.4%, *P* < 0.001). In satellite tumors, activation levels were similar across groups. After Cryo + CpG, treated tumors had higher early CTL activation than satellite tumors (40.5% difference, *P* < 0.001; Supplement Fig. 3d).

While CD69 marks transient activation, sustained T cell activity is indicated by CD137 expression [27]. All CPI-treated groups showed decreased CD137 + CTLs versus Control in both treated and satellite tumors, while CpG alone had no effect (Fig. 3e). Three-way ANOVA identified CPI as the dominant factor, accounting for 53.3% and 53% of the variance in treated and satellite tumors, respectively (*P* < 0.001; Supplementary Fig. 2g, h). Pooled analysis confirmed CPI-driven decreases (− 4.9% and − 5.4%; both *P* < 0.001; Supplementary Fig. 3f).

CpG + CPI showed higher Th frequencies versus Control in both treated and satellite tumors, with mean differences of 9.2% (*P* = 0.008) and 9.4% (*P* = 0.011), respectively (Fig. 3b).

### CPI or combination therapies increases effector CTLs, while CpG + CPI (± Cryo) enhances effector-to-naïve CTL and Th ratios

Effector T cell frequencies were assessed to elucidate the impact of these treatments on cell-mediated immunity. Effector CTL and effector Th cell frequencies were assessed using CD44 and CD62L as markers for naive (CD44-, CD62L +), effector (CD44 + , CD62L-), or central memory cells (CD44 + , CD62L +) [28]. Effector CTLs (Fig. 3f, i) were increased in parallel to total CTL (Fig. 3a). CPI-containing treatments increased effector CTL frequencies in both treated and satellite tumors. Three-way ANOVA identified CPI as the dominant driver, accounting for 39.8% and 55.7% of the variance, respectively (*P* < 0.001; Supplementary Fig. 4a, b). Pooled analysis confirmed CPI-driven increases (13% and 14.7%; *P* = 0.001 and *P* < 0.001; Supplementary Fig. 4i).

Effector Th cell frequencies were similar across groups (Supplementary Fig. 4j), but three-way ANOVA revealed a significant CPI effect in treated tumors (37.12% variance, *P* = 0.004; Supplementary Fig. 4c). Pooled analysis confirmed CPI-driven increases in treated lesion (10.1%, *P* = 0.002; Supplementary Fig. 4k).

While absolute effector T cell frequencies indicate immune activation, the effector:naïve ratio more closely reflects functional status. This ratio was assessed for CTL and Th cells across groups. CpG + CPI showed higher ratios in both tumor sites (Fig. 3g, h), with similar trends in Th cells.

### CpG + CPI abrogates Cryo-induced PD-1 upregulation and Cryo + CpG enhances PD-1/ CTLA-4 co-expression

**Fig. 4:**
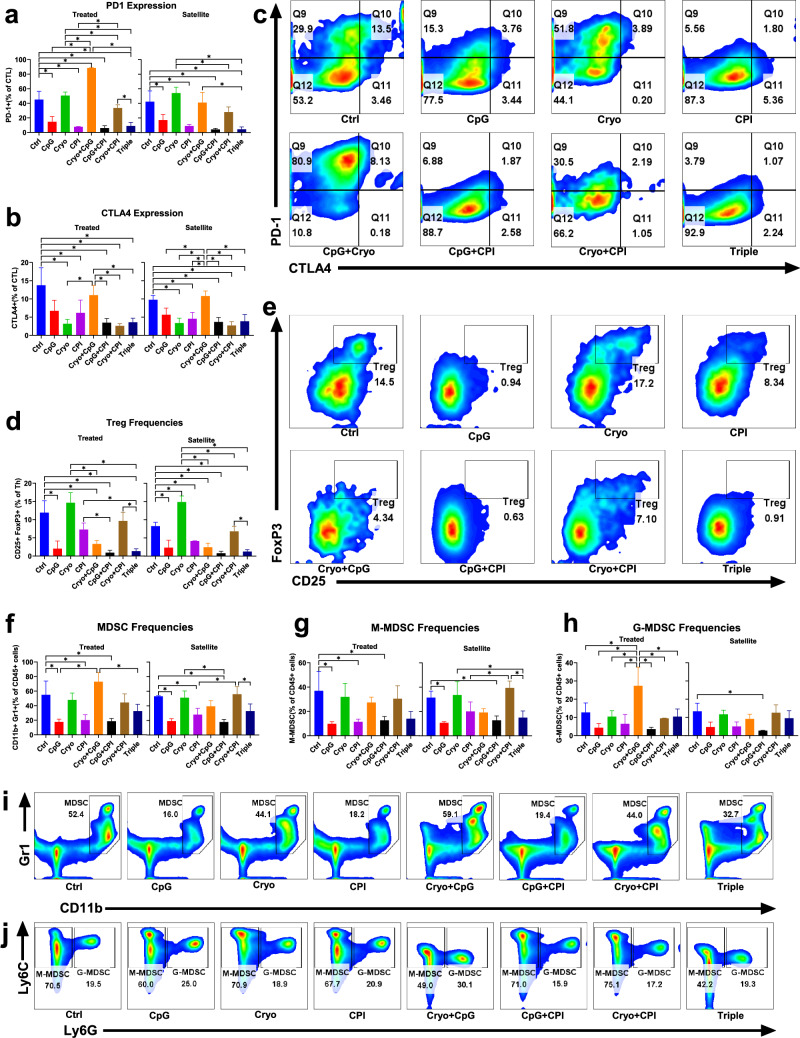
Combination therapies modulate immunosuppressive cell populations and reduce T cell exhaustion. **a** PD-1 expression on CTLs analyzed across groups, CpG or CPI monotherapy reduced expression of PD-1 versus Control in both treated and satellite tumors, with mean differences of 30.5% (*P* < 0.001) and 37.3% (*P* < 0.001), 25.2% (*P* = 0.044) and 33.3% (*P* = 0.005), respectively. Cryo + CpG + CPI lowered PD-1 expression versus Control in both treated and satellite tumors, with mean differences of 36.3% and 37.8% (both *P* ≤ 0.001), respectively. **b** CTLA-4 expression on CTLs analyzed across groups. **c** Representative PD-1 and CTLA-4 flow cytometry plots from treated tumors. **d** CD25^+^FoxP3^+^ Tregs (within CD4^+^ T cells) analyzed across groups. **e** Treg frequencies compared between pooled CpG-treated and non-CpG-treated cohorts. **f** Total MDSCs (CD11b^+^Gr1^+^) analyzed across groups in treated and satellite tumors. **g**–**h** Frequencies of M-MDSCs (CD11b^+^Gr1^+^Ly6G⁻Ly6C^+^, **g**) and G-MDSCs (CD11b^+^Gr1^+^Ly6G^+^Ly6C^low^, **h**) across groups. **i**–**j** Representative flow cytometry plots showing MDSC gating strategies: CD11b/Ly6G for total MDSCs (i), and Ly6G/Ly6C for MDSC subsets (j). (*P < 0.05)

Given differences in CTL frequency, activation, and effector-naïve balance across treatments, immune checkpoint regulation was next assessed. CpG and CPI monotherapy reduced PD-1 expression versus Control in both treated and satellite tumors (Fig. 4a, c), while Cryo alone had no effect and eliminated the effect of CpG or CPI when combined. However, the Triple therapy restored this reduction. Three-way ANOVA (Supplementary Fig. 5a, b) identified CPI and Cryo as strong contributors to PD-1 regulation in treated tumors (42.4% and 24.3% variance, both *P* < 0.001), with CPI remaining dominant in satellite tumors (49.3%, *P* < 0.001).

CTLA-4 expression was assessed across groups. In both treated and satellite tumor, CTLA-4 expression was significantly higher in the Control group compared to all other groups, except CpG and Cryo + CpG (Fig. 4b, c). Cryo + CpG group exhibited the highest CTLA-4 expression among all combination treatment groups. Three-way ANOVA (Supplementary Fig. 5c, d) identified CPI as strong contributors to CTLA-4 regulation both treated and satellite tumors (28.9% and 35.3% variance, both *P* < 0.001).

PD-1/CTLA-4 co-expression was assessed to explore immunoregulatory interactions. Cryo + CpG markedly increased co-expression compared to all other groups except for Control group (Supplementary Fig. 5i).

### CpG-related treatments reduce Treg frequencies

Beyond CTL responses and checkpoint regulation, regulatory T cells (Tregs) play a crucial role in immune suppression [29]. Treg frequencies were assessed across groups (Fig. 4d, e). CpG-containing regimens (CpG, Cryo + CpG, CpG + CPI, Triple) significantly reduced Tregs compared to Control in both treated and satellite tumors. Cryo alone increased Tregs in satellite tumors versus Control (*P* = 0.001), but the effect in treated tumors was not significant. No significant differences in Treg frequencies were observed between treated and satellite tumors within each group (Supplementary Fig. 6c). Three-way ANOVA confirmed strong CpG effects on Treg frequencies in both tumor sites (Supplementary Fig. 6a, b). Pooled analysis confirmed CPI-driven decreases (Supplementary Fig. 6d).

### Unsupervised clustering reveals distinct T cell subset dynamics across treatment conditions

**Fig. 5:**
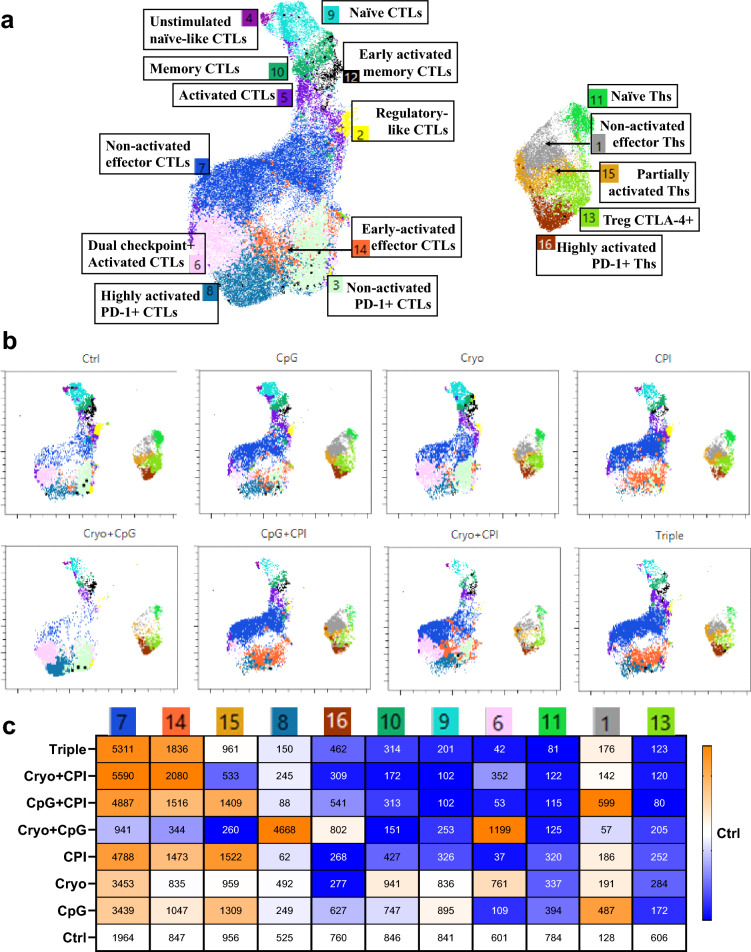
Unsupervised clustering reveals distinct T cell activation and differentiation states across treatment groups. **a** Unsupervised clustering of pooled CD4^+^ and CD8^+^ T cells from all treatment groups, visualized by PaCMAP. Clusters are annotated by immunophenotype based on expression of T cell markers. Left: CTL subsets; Right: Th subsets. **b** Cluster overlays shown by treatment group. **c** Cluster abundance heatmap across treatments, normalized to Control. Orange indicates enrichment; blue indicates reduction. Only clusters with ≥ 500 cells in at least one group are shown

To further characterize these tumor-infiltrating T cell populations, we applied unsupervised clustering to CD45 + cells stained with the T cell panel (CD4, CD8, CD44, CD62L, CD69, CD137, PD-1, CTLA-4, FoxP3, CD25) from all treatment groups (Supplementary Fig. 7a–c). This initial clustering revealed a large population of CD45^+^CD4⁻CD8⁻ cells, likely representing MDSCs or other myeloid populations. Given our focus on T cell subsets, we reclustered the CD4^+^ and CD8^+^ T cells across groups (Fig. 5a, b; marker heatmap in Supplementary Fig. 8e).

Three major T cell clusters were enriched in CPI-treated groups, clusters 7, 14 and 15. Cluster 7, the most abundant population (38%; Supplementary Fig. 8e), consisted of effector CTLs lacking activation (CD69⁻CD137⁻) and checkpoint (PD-1⁻CTLA-4⁻) markers. These may reflect checkpoint-released, previously exhausted CTLs. All CPI-containing treatments showed increased Cluster 7 cells (Fig. 5b, c), while Cryo + CpG showed fewer than control. Cluster 14 included early-activated effector CTLs (CD69^int^, PD-1⁻CTLA-4⁻) and was similarly enriched in CPI-treated groups but reduced in Cryo + CpG. These cells may reflect newly recruited CTLs in early activation, or checkpoint-released effectors re-engaging their functional program. Cluster 15, composed of partially activated Ths (CD137^int^, PD-1⁻CTLA-4⁻), was also elevated in some CPI-treated conditions.

Cryo treatment increased newly recruited CTLs (cluster 7) and decreased naive and activated effector Th cells (clusters 8 and 16) and Tregs (cluster 13); CpG treatment increased newly recruited CTLs and decreased activated CTLs (clusters 8 and 6) and Tregs (cluster 13); In contrast, the combined Cryo plus CpG treatment greatly increased activated CTLs (clusters 8 and 6). The triple combination treatment most closely resembled the CPI treated groups, suggesting that CPI had the greatest effect on T-cell populations.

### CpG and CPI reduce MDSC levels in Cryo-treated tumors; PD-1 expression correlates positively with MDSC frequencies.

Immunosuppressive myeloid cells such as MDSCs also regulate the TME and influence treatment outcomes [30]. To assess their role, MDSC frequencies were analyzed across groups. CpG and CPI alone and together reduced MDSC frequencies in treated and satellite tumors compared to Control (Fig. 4f, i). Cryo alone had no effect and negated the reductions seen with CpG or CPI. Three-way ANOVA confirmed Cryo effects in both tumors (Supplementary Fig. 9a, b), CPI in treated tumors, and CpG in satellite tumors. PD-1 expression correlated with MDSC levels (ρ = 0.768, *P* < 0.001).

MDSCs were subdivided into monocytic (M-MDSCs) and granulocytic subsets. M-MDSC trends mirrored total MDSCs in both treated and satellite tumors (Fig. 4g, j). Three-way ANOVA identified CpG as the dominant factor in satellite tumors (56.4% of the variance, *P* < 0.001; Supplementary Fig. 9d). Granulocytic MDSCs (G-MDSCs) were elevated in treated but not satellite tumors in Cryo + CpG compared to all other groups (Fig. 4h, j).

### Correlation between circulating and tumor-infiltrating immune populations and treatment-induced modulations in peripheral blood

**Fig. 6:**
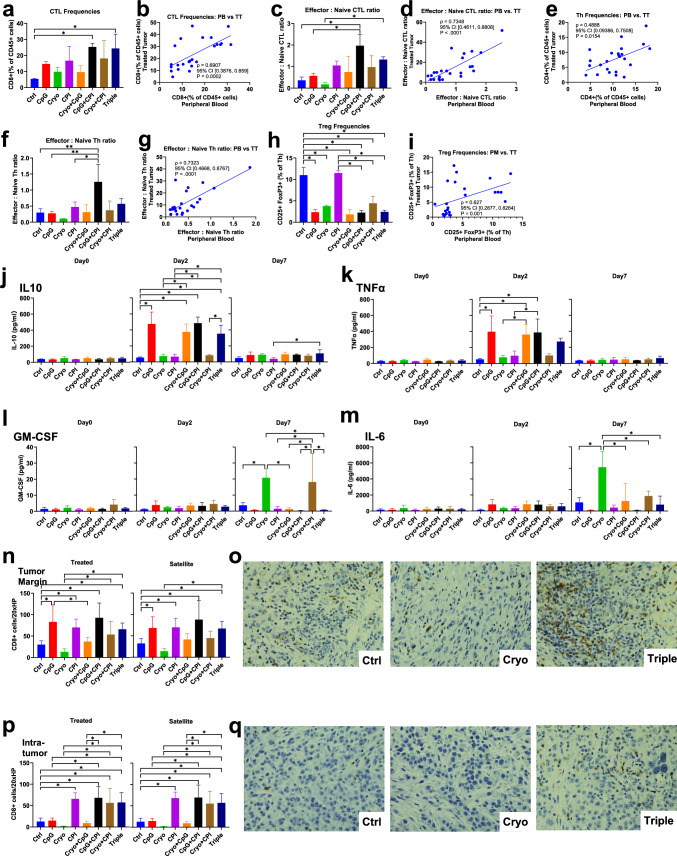
Peripheral and intratumoral immune responses reveal coordinated systemic and local T cell activation across treatments. **a**, **c**, **f**, **h** Quantification of circulating total CTL frequencies (**a**), effector:naïve CTL ratio (**c**), effector:naïve Th ratio (**f**), and Treg frequencies (**h**) across treatment groups. **b**, **d**, **e**, **g**, **i** Correlation of matched peripheral blood (PB) and treated tumor (TT) samples for total CTLs (**b**), effector:naïve CTL ratio (**d**), Th frequencies (**e**), effector:naïve Th ratio (**g**), and Treg frequencies (**i**). **j**, **k**, **l**, **m** Serum levels of IL-10 (**j**), TNF-α (**k**), GM-CSF (**l**), and IL-6 (**m**) measured on Days 0, 2, and 7 across treatment groups. **n**, **p** Quantification of CD8^+^ T cells per 20 × HPF in the tumor margin (**n**) and intra-tumor (**p**) regions of treated and satellite tumors. **o**, **q** Representative IHC images showing CD8^+^ T cells (brown) in the tumor margin (**o**) and intra-tumor (**q**) for Control, Cryo, and Triple therapy groups. (*P < 0.05; PB = peripheral blood; TT = treated tumor)

To assess systemic immunity, we analyzed peripheral blood T cells and their correlation with tumor-infiltrating populations. Circulating CTLs increased in CpG + CPI and Triple groups (Fig. 6a), correlating with intratumoral CTLs (Fig. 6b). Effector CTLs (CD44^+^CD62L⁻) were elevated in all CPI-treated groups (Supplementary Fig. 10a). The effector/naïve CTL ratio was highest in the CpG + CPI group and correlated with tumor ratios (Fig. 6c, d).

Peripheral Th frequencies were not changed (Supplementary Fig. 10c), but correlated with intratumoral Th frequencies (Fig. 6e). Th effector Ths were only elevated with CpG + CPI and did not correlate with intratumoral effector Th frequencies (Supplementary Fig. 10d, e). Similarly, the effector/naive ratios in Th cells were elevated after CpG + CPI and did correlate with intratumoral ratios (Fig. 6f, g). Tregs were suppressed by CpG and Cryo but not CPI, were also suppressed by any combination therapy and correlated with intratumoral Th frequencies (Fig. 6h, i). Pooled analysis confirmed lower Treg frequencies in CpG-treated groups than in non-CpG-treated groups (Supplementary Fig. 10h).

### Plasma Cytokine levels over time

To further explore how these treatments influence anti-tumor immunity, systemic cytokine responses were analyzed by measuring plasma cytokine levels at baseline (Day 0), early (Day 2), and late (Day 7) time points post-treatment. All CpG-treated groups exhibited higher IL-10 and TNFα levels compared to Control on Day2 post-treatment, with levels decreasing across all groups by 1 week (Fig. 6j, k).

Plasma IL-10 and TNF-α levels on Day 2 showed an inverse exponential correlation with Treg frequencies in treated tumors (Supplementary 11d, e). At 1-week post-treatment, the Cryo group demonstrated higher GM-CSF and IL-6 levels than Control (Fig. 6l, m). Day7 plasma IL6 levels were elevated if samples were categorized into Cryo-treated and non-Cryo-treated groups (Supplementary Fig. 11h).

#### Histologic analysis reveals distinct patterns of CTL infiltration across tumor compartments

CD8^+^ T cell infiltration in the tumor margins, the tumor, and adjacent normal liver tissue was assessed by immunohistochemistry in both treated and satellite tumors. Compared to Control, CpG and CPI increased CTL infiltration in the tumor margin in both treated and satellite tumors whereas Cryo did not (Fig. 6n, o). Intratumoral CTLs increased in all CPI-containing groups in both treated and satellite tumors as compared to Control (Fig. 6p, q). Liver CTLs showed a similar pattern of infiltration (Supplementary Fig. 11i).

## Discussion

This study demonstrates that Cryo combined with intratumoral immune stimulation with CpG and dual CPI according to the STRIDE regimen inhibited the growth of both treated and satellite HCC tumors, promoted a more immunogenic TME, and prolonged survival. Whereas the Triple therapy induced systemic anti-tumoral immunity (i.e. abscopal effect), partial Cryo alone induced systemic immunosuppression, promoting satellite tumor growth and a more immunosuppressive TME.

Cryo depleted CTLs in the tumor and surrounding liver but increased CTLs at the tumor margins, characteristic of an immune-excluded tumor phenotype. The Cryo-induced increase in Tregs, particularly in the satellite tumor, further contributes to an immunosuppressive TME. Immune stimulation, either locally with CpG or systemically with CPI, increased both circulating and intratumoral CTLs, reduced both treated and satellite tumor size, and improved survival.

Cryo + CpG + CPI suppressed growth of both treated and satellite tumors and relieved the immunosuppressive TME by decreasing checkpoint proteins, Tregs, and MDSCs, and by increasing total, activated and effector CTLs. CPI proved to be a major driver of CTL recruitment and effector differentiation, whereas CpG was the major driver of Treg depletion. While Tregs uniformly expressed CTLA-4, their depletion appeared to be primarily driven by CpG rather than by dual checkpoint blockade. Despite the anti-tumor benefits of CpG and CPI, alone or in combination, the Triple therapy yielded the most profound effects on survival and TME. Cryo + CpG + CPI conferred a durable survival benefit, with 50% of these mice surviving 120 days post-treatment compared to only 6 days for Control. The strength of the triple combination likely reflects the additive contributions of each component. Cryo releases TAAs and DAMPs, which are subsequently processed and presented by APCs [31]. Previous studies have shown that CpG, a TLR9 agonist, amplifies APC activation, leading to increased cytokine secretion (e.g., IL-12, IFN-α/β) [32]; this may contribute to the sustained CD69 expression observed after Cryo + CpG. This cytokine-rich environment, combined with feedback from IFN-γ–producing CTLs and Th1 cells, may reinforce early T cell activation within treated tumors [33]. In contrast, satellite tumors do not receive direct local stimulation from Cryo or CpG and do not exhibit the increased CD69 expression characteristic of early lymphocyte activation. Sustained CD69 expression is not necessarily beneficial, as it may reflect a stalled immune state characterized by prolonged early activation without effector differentiation [34]. Notably, the addition of CPI to Cryo + CpG normalized CD69 levels, suggesting that checkpoint blockade facilitates the transition from early CTL activation to full effector function. Indeed, higher effector CTL frequencies were observed in all CPI-containing groups, highlighting CPI’s critical role in promoting T cell differentiation and functional maturation.

CpG and CPI monotherapies reduced PD-1 expression, whereas Cryo—alone or in combination with either agent—did not. Notably, in the Triple Therapy group, PD-1 expression remained suppressed at levels similar to CpG or CPI monotherapy, suggesting that combined CpG and CPI can overcome the immunosuppressive effect of Cryo.

In addition to T cell-mediated immunity and Treg suppression, our study highlights the role of immunosuppressive MDSCs in shaping treatment responses. The observed positive correlation between PD-1 expression and MDSC frequencies supports a potential interplay between immune checkpoint regulation and myeloid-driven immunosuppression within the TME [35]. Cryo appears to strengthen this immunosuppression with the stimulation of pro-inflammatory cytokines such as IL-6, GM-CSF, and VEGF [31]. Indeed, this study showed that Cryo induced higher levels of GM-CSF and IL-6 than most other groups at 1-week post-treatment, which may promote the recruitment, activation, and expansion of MDSCs [30].

MDSCs, in turn, suppress anti-tumor immunity by producing factors like IL-10, TGF-β, and reactive oxygen species (ROS), which inhibit CTLs and promote their exhaustion [30]. Exhausted CTLs express higher PD-1 which may bind to PD-L1 and enhance MDSC survival and proliferation [36], forming a feedback loop that sustains immunosuppression. Additionally, exhausted CTLs produce immunosuppressive cytokines such as IL-10 and VEGF, further supporting MDSCs and their suppression of anti-tumor immune responses [37].

**Fig. 7:**
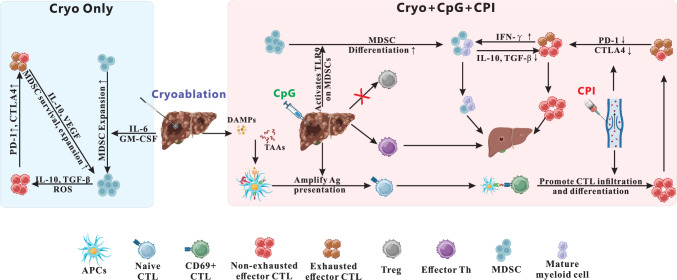
Schematic model of proposed immunological effects: Cryo vs. Cryo + CpG + CPI. Left (blue panel): Cryo induces TAA release and inflammatory cytokines (e.g., IL-6, GM-CSF, VEGF), driving MDSC recruitment and expansion. MDSCs suppress CTLs via IL-10, TGF-β, ROS, and promote T cell exhaustion. PD-1 expression on CTLs enhances MDSC survival through PD-1/PD-L1 signaling, creating a feedback loop of immunosuppression. Right (red panel): Cryo + CpG + CPI remodels the tumor microenvironment. CpG boosts TAA presentation, MDSC differentiation and surpresses Treg; CPI reactivates exhausted CTLs and promotes effector function. The combination lowers PD-1/CTLA-4 expression, reduces MDSCs and Treg, and enhances effector CTL infiltration and anti-tumor immunity

The proposed mechanisms supported by these results are summarized in Fig. 7. Cryo alone sheds TAA that could be immunostimulatory, yet it maintains an immunosuppressive state by inducing GM-CSF and IL6 and preventing PD1 downregulation. Overcoming this immunosuppression requires two effects: (1) CpG to reprogram the TME, enhance antigen presentation and T cell activation, and suppress MDSCs and Tregs and (2) dual CPI (anti PD-1 and anti-CTLA-4) to relieve checkpoint-mediated CTL suppression and enhance T cell function. Together, they effectively transform the immunosuppressive TME into one more conducive to anti-tumor immunity.

Future studies incorporating targeted immune-cell depletion and antigen-tracking approaches will be essential for determining whether the observed treatment effects are truly additive or synergistic. Notably, in this murine model, the smallest cryoablation ice ball technically achievable produces an ablation zone that is disproportionately large relative to the size of the mouse liver, leading to substantial antigen and DAMP release and pronounced myeloid-driven immunosuppression. This strong suppressive effect may mask any potential synergy between treatment components. In addition, functional validation through CD8^+^ T-cell depletion and antigen-specificity assays will be necessary to directly confirm the causal role of T cells in mediating tumor regression. Finally, future work will extend these findings across additional HCC and syngeneic tumor models in both male and female mice to confirm the generalizability of the observed immune and therapeutic responses.

In summary, our study highlights the distinct and complementary roles of CpG and CPI combined with Cryo in reshaping the TME and enhancing local and systemic antitumor immunity. Future preclinical studies focusing on refining the timing, dosing, and sequencing of Cryo, CpG, and CPI therapies could maximize antitumor responses while minimizing immunosuppressive rebound and immune toxicities. A phase 1 clinical trial (NCT06710233) is currently underway assessing the safety and tolerability of partial cryoablation with transarterial hepatic administration of CpG (nelitolimod) one week prior to initiating the STRIDE regimen of dual CPI in patients with advanced HCC. If human studies align with murine studies, this Triple therapy could improve upon the current 20% response rate to STRIDE and promote the durable, systemic antitumor immunity needed to prolong survival and reduce the high recurrence rates for HCC.

## Supplementary Information

Below is the link to the electronic supplementary material.

Supplementary file 1 (DOCX 9459 KB)

## Data Availability

The datasets generated during and/or analyzed during the current study are available from the corresponding author on reasonable request.
