# Supplementary material for: Stoking an anti-liver cancer immune response with cryoablation plus an intratumoral TLR9 agonist and dual checkpoint inhibitors
Source: Cancer Immunol Immunother. 2026 Feb 12;75(3):74. doi: 10.1007/s00262-025-04286-8 (PMC12901810; doi:10.1007/s00262-025-04286-8)
Supplement: Supplementary file 1 — (DOCX 9459 KB) [file 262_2025_4286_MOESM1_ESM.docx]

**Supplementary Materials and Methods:**

**Animal Studies:**

The initial sample size of 7 mice per group was determined using a power analysis performed with G*Power 3.178, based on an effect size of 0.80 derived from preliminary data, an alpha error probability of 0.05, and a power of 0.85. To account for an anticipated 15% mortality rate due to disease progression or complications, 3 more mice were included per group, increasing the adjusted total to 10 mice per group. Furthermore, 3 additional mice per group were allocated for histologic and flow cytometric analyses, resulting in a final total of 13 mice per group. This study utilized 104 male C57BL/6J mice (Jackson Laboratory). Breeding colonies were housed in individually ventilated cages under a 12-hour light/dark cycle, with lights on at 6:00 AM. A maximum of four littermate mice of the same sex were group-housed per cage starting at weaning. Spruce wood shavings were used as bedding material. At 8 weeks of age, the mice were placed on a western diet (D12079, Research Diets, New Brunswick, New Jersey) to induce metabolic dysfunction-associated steatohepatitis (MASH).

After 4 weeks mice,mice were randomized into one of 8 treatment groups using a random number generator. To create a model of HCC, mice were implanted with RIL-175 cells, an HCC cell line developed in C57BL/6 mice by transplanting HRasV12-transduced p53−/− fetal hepatoblasts (Zender et al., 2005). Isoflurane anesthesia was administered via a nose cone, and the abdominal areas were shaved and sanitized with iodine solution. To establish the tumor model, two orthotopic HCC foci were created percutaneously using a 70-MHz ultrasound (US) transducer with the Vevo MD Ultra High-Frequency US System (VisualSonics, Fujifilm; Ontario, Canada) to guide the coaxial injection of RIL-175 cells into right and left liver lobes. Each site received an injection of 1 × 10⁶ RIL-175 cells suspended in 25 μL of Cultrex Basement Membrane Extract (Sigma-Aldrich; Burlington, Massachusetts), delivered through a 22-gauge needle. One site served as a “satellite” tumor for observation, while the other was designated for treatment.

**Treatment Interventions**

Each group received the following prescribed treatments or a mock/sham treatment starting on Day 0, with additional CPI doses delivered at intervals to simulate the STRIDE regimen:

- **Control (Ctrl)**: Receive mock CpG and mock CPI plus a sham procedure.
- **CpG only**: Receive CpG and mock CPI and a sham procedure.
- **Cryo only**: Undergo Cryo with mock CpG and mock CPI.
- **CPI only**: Receive CPI plus mock CpG and a sham procedure.
- **Cryo+CpG**: Undergo Cryo and CpG with mock CPI.
- **Cryo + CPI**: Undergo Cryo and CPI with mock CpG.
- **CpG+CPI**: Receive CpG and CPI and a sham procedure.
- **Cryo+CpG+CPI** or **Triple Therapy**: Cryo plus intratumoral CpG and systemic CPI.

**CpG:** 100 μg of CpG ODN 2395, a type C CpG (IAX-200-007-M001; Adipogen, San Diego, California), diluted in 50 μl 0.9% endotoxin-free saline, was injected in 3 fractions along the tumor ablation margins or in the tumor under US guidance. For the mock CpG, the same volume of saline was given under identical conditions.

**CPI:** 125 µg of InVivoMAb anti-mouse PD-1 antibody and 250 µg of InVivoMAb anti-mouse CTLA-4 (CD152) antibody were delivered by intraperitoneal injection on Day 0 and anti-PD-1 antibody was re-administered every 3 days for 2 weeks total. For the mock CPI, an equal volume of 0.9% endotoxin-free saline was delivered under identical conditions.

**Cryo:** the Galil cryoablation system (Boston Scientific) with a 17G, 3-cm IceSeed probe was used to perform three cycles of one-minute freezes at 50% power to achieve incomplete (75% or less) tumor ablation. For the sham Cryo, the cryoprobe was inserted for the same duration but without activating it.

For general surgeries and implantations: Mice are placed on a Deltaphase isothermal heating pad until they become mobile at which time they are transferred to a clean cage. Mice from cryoablation procedure will be dried off with gauze then placed on Deltaphase pad.

Periprocedural Care: Anesthesia and measures to limit animal pain and distress: General anesthesia with isoflurane (3% induction in induction box; 1.5% maintenance, 2 L/min O2 via nasal cone) will be used for ultrasound assessments of the liver tumors and for percutaneous interventions. For cryoablation, local anesthesia with lidocaine 1% will also be used. Analgesics will be given before cryoablation. Body temperature will be maintained by Deltaphase heating pads. cryoablation will be performed using aseptic technique (sterile instruments and gloves, aseptic preparation of the surgical field). In all animals, the depth of anesthesia will be monitored, assessing movement on tail pinch and respiratory rate changes.

Post-procedural care: Immediately after procedure, the animals will be monitored by lab personnel until they wake and for the first 3 hours or until their activity returns to normal. Weights will be recorded weekly or up to every other day as tumors advance. In addition to assessing for weight loss, other indices of health that will be noted, including decreased appetite, impaired ambulation, tachypnea, signs of infection, lethargy, ruffled fur, hunched posture, bleeding, central nervous system disturbances, diarrhea or constipation, or muscle atrophy. Any animals showing any of these signs will be reported to the ACP and their recommendations will be followed. Animals found to be moribund will be euthanized.

Based on prior experience with the tumor models and procedures employed in this study, the animals did not exhibit overt signs of pain, distress, or impaired welfare that would necessitate the routine use of analgesics. However, analgesics (buprenorphine) were readily available and would have been administered if animals had shown any clinical signs of discomfort, including reduced mobility, vocalization, abnormal posture, or reduced food/water intake. Animals were monitored daily for such signs, and the protocol allowed for immediate veterinary intervention if needed. This approach was approved by the Institutional Animal Care and Use Committee (IACUC), considering both animal welfare and the potential confounding effects of analgesic agents on immune responses and tumor progression.

**Tumor Volume Characterization and Endpoints**

Tumors were assessed using US by an assessor who was blinded to the intervention, the largest dimensions of viable tumors measured in both the axial and sagittal planes. Tumor volumes were determined using the formula: Volume = π/6 * (x * y * z), where x represents the left-to-right diameter, y is the craniocaudal diameter, and z is the anteroposterior diameter. Only tumors that were 5 mm or larger were considered for treatment. At the time of treatment, the sizes of the tumors were statistically similar across all groups. To account for initial heterogeneity in tumor volumes, fold changes in tumor volumes were calculated by comparing the volumes one week after treatment to the volumes at the start of treatment.

The criteria for sacrificing mice in the time-to-endpoint analysis included tumors exceeding 1 cm in any dimension or mice exhibiting difficulty in ambulating, poor grooming, weight loss, or visible injuries. 3 mice of each group are sacrificed at 1week post-treatment for flow cytometry and histology studies. Peripheral blood was collected for flow cytometry at the same time. Fig. 11 contains additional details of study design, including subject grouping and exclusion process.

**Flow Cytometry and Histology**

Seven days after the initiation of treatment, 3 mice per group were euthanized using isoflurane anesthesia followed by cervical dislocation for flow cytometry and histology analysis. Tumors were bisected; one half was fixed in 4% formaldehyde in PBS, processed, and submitted to the UC San Diego Moores Cancer Center Biorepository and Tissue Technology Shared Resource for paraffin embedding, thin sectioning, hematoxylin and eosin (H&E) staining, and immunohistochemistry (IHC). IHC was performed using a primary antibody against murine CD8 (Cat# ab183685; Abcam).

The remaining tumor half and peripheral blood were processed for flow cytometry. Tumor tissues were minced and digested in 1% collagenase PBS solution at 37°C for 30 minutes, followed by filtration through a 100-μm cell strainer (Cat# 877119, Thermo Fisher Scientific, Waltham, Massachusetts). Red blood cells (RBCs) were lysed using ACK Lysing Buffer. Immune cells were subsequently enriched via a 40%-70% Percoll gradient centrifugation at 1000×g for 30 minutes with the brake off. Immune cells were collected from the interface between the 40% and 70% layers.

For flow cytometry analysis, cells were divided into two panels: one for T cell analysis and one for myeloid cell analysis (see below). Peripheral blood samples were processed exclusively for the T cell panel. Unstained cells, single stain, fluorescence minus one (FMO) controls, and isotype controls were used for instrument compensation, gating strategy validation, and to ensure specificity of antibody staining. Data were analyzed using FlowJo software (FlowJo, Ashland, Oregon).

T cell panel

| Antibody | Clone | Cat# |
| --- | --- | --- |
| CD279 | 29F.1A12 | Biolegend 135213 |
| CD137 | 17B5 | Biolegend 106105 |
| CD44 | IM7 | Biolegend 103056 |
| CD62l | MEL-14 | Biolegend 104410 |
| CD152 | UC10-4B9 | Biolegend 106314 |
| FoxP3 | MF-14 | Biolegend 126408 |
| CD25 | PC61 | Biolegend 102026 |
| CD69 | H1.2F3 | Biolegend 104545 |
| Zombie Viability Kit |  | Biolegend 423102 |
| CD4 | RM4-5 | Biolegend 100548 |
| CD45 | 30-F11 | Biolegend 103151 |
| CD8a | 53-6.7 | Biolegend 100750 |

Tab. 1

Myeloid cell panel

| Antibody | Clone | Cat# |
| --- | --- | --- |
| F4/80 | BM8 | Biolegend 123110 |
| CD45 | 30-F11 | Biolegend 103132 |
| Ly6G | 1A8 | Biolegend 127614 |
| CD11b | M1/70 | Biolegend 101236 |
| Gr1 | RB6-8C5 | Biolegend 108442 |
| Ly6C | HK1.4 | Biolegend 128041 |
| Zombie Viability Kit |  | Biolegend 423102 |

Tab. 2

**Chemokine/Cytokine Analysis**

Tail bleed samples (50 µL) were collected from four mice per group at predetermined time points—Days 0, 2, and 7—using tubes containing ethylenediaminetetraacetic acid (EDTA) as an anticoagulant. Following collection, the samples were centrifuged at 6,000 rpm for 6 minutes at 4°C to separate the plasma. The resulting supernatant was stored at −80°C until further analysis.

The chemokine and cytokine profiles were quantitatively analyzed using an electrochemiluminescence-based multiplex immunoassay on the MESO QuickPlex SQ 120MM system (Meso Scale Diagnostics, Rockville, Maryland). The assay targeted key analytes, including GM-CSF, IL-2, IL-6, IL-10, CXCL1, TNF-α, IL-12p70, IL-13, and IL-4 using the U-PLEX Custom Biomarker Group 1 Assays (Product #K15069M-1, Meso Scale Diagnostics). All procedures were performed in strict accordance with the manufacturer's protocols. The levels of IL-12p70, IL-13, and IL-4 were below the detection limit of the ELISA kit.

**Data Analysis and Statistical Methods**

All statistical analyses were performed using Prism 10 (GraphPad, San Diego, California). Fold volume changes of tumors and immune parameters measured by flow cytometry were analyzed using one-way ANOVA, with post hoc Tukey’s multiple comparisons test to compare groups. Analyses were performed separately for treated and satellite tumors. Differences between treated and satellite tumors within each group were evaluated using two-way ANOVA, followed by Šídák’s multiple comparisons test to adjust for multiple testing. Three-way ANOVA was used to assess the independent and combined effects of Cryo, CpG, and CPI on tumor growth and immune parameters, including main effects and interactions, with Tukey’s multiple comparisons test applied for post hoc group comparisons. Survival data were analyzed using the Kaplan–Meier method. Survival curves were generated for each group and compared using the log-rank (Mantel–Cox) test to determine statistically significant differences in survival distributions.

Correlation analyses were performed to assess the relationships between immune parameters in peripheral blood and tumors, as well as between systemic cytokines and intratumoral immune features. Spearman’s rank correlation coefficient (ρ) was used to evaluate nonparametric associations between variables. Nonlinear regression analysis was applied where appropriate, and fitted curves were generated to model the relationship between plasma cytokine levels and intratumoral Treg frequencies. The strength of correlations was reported using Spearman’s ρ values, associated *P* values, and 95% confidence intervals. Goodness of fit for nonlinear models was evaluated by the coefficient of determination (R²).

**Figure. 1:**

(a) In treated tumors, CPI and CpG accounted for 25.73% and 20.80% of the total variance, respectively (both *P* < 0.001), while Cryo contributed a smaller effect (4.04%, *P* = 0.032). A modest interaction between CpG and CPI was observed (5.75%, *P* = 0.011). No significant three-way interaction (Cryo × CpG × CPI) was detected.

(b) Satellite tumors showed a similar pattern: CpG and CPI accounted for 27.37% and 18.59% of the variance, respectively (both *P* < 0.001), with Cryo exerting a smaller effect (3.07%, *P* = 0.045). Two interaction effects reached statistical significance: Cryo × CpG (10.33%, *P* < 0.001) and CpG × CPI (3.22%, *P* = 0.040). The three-way interaction was not significant.

(c) Comparison of fold volume change between treated and satellite tumors across different groups at 1-week post-treatment. Cryo alone or with CPI led to a significant increase in satellite tumor growth compared to their respective treated tumors 1-week post-treatment.
(In all panels, ‘T’ indicates treated tumors and ‘S’ indicates satellite (untreated) tumors)
(**P* < 0.05)

**Figure. 2**

(a, b) Three-way ANOVA of CTL in treated and satellite tumors. CPI was the principal contributor to variation in CTL frequencies, accounting for 77.17% and 85.54% of the total variation in treated and satellite tumors, respectively (*P* < 0.001). In treated tumors(a), neither Cryo nor CpG alone affected CTL frequencies, whereas in satellite tumors, CpG exerted a modest main effect (3.14% of variation, *P* = 0.019), while Cryo was not significant. In treated tumors, two significant but modest interaction effects were observed: Cryo × CpG (6.88%, *P* = 0.003) and CpG × CPI (4.80%, *P* = 0.01). No significant three-way interaction among Cryo, CpG, and CPI was detected in either treated or satellite tumors.

(c, d) Three-way ANOVA of Th in treated and satellite tumors. Cryo and CPI were the principal contributors to variation in CD4⁺ T cell frequencies, accounting for 28.14% and 22.9% of the total variation in treated tumors, and 26.3% and 28.32% in satellite tumors, respectively (*P* < 0.001). In both tumor sites, CpG alone did not significantly impact CD4⁺ T cell frequencies. In treated tumors (Fig. b), a modest but statistically significant three-way interaction among Cryo, CpG, and CPI was detected (7.87% of variation, *P* = 0.0358), whereas all pairwise interaction terms were not significant. In satellite tumors (Fig. c), a significant interaction between Cryo and CPI was observed (8.39%, *P* = 0.0318), as well as a three-way interaction (8.19%, *P* = 0.0337), suggesting combinatorial effects of therapy components on CD4⁺ T cell infiltration.

(e, f) Three-way ANOVA of early activated CD69^+^ CTL revealed statistically significant, yet modest, main effects of Cryo (14.65%), CpG (15.76%), and CPI (13.69%) on early activated CTL frequencies in treated tumors (*P* < 0.001 for all), as well as significant two-way (Cryo × CpG, 19.18%; Cryo × CPI, 5.96%; CpG × CPI, 6.27%) and three-way (16.37%) interaction effects. Notably, although these effects were statistically significant, each accounted for a relatively limited portion of the total variation (<20%), suggesting that no single treatment or interaction solely dominated the early CTL activation landscape. In satellite tumors, three-way ANOVA identified significant weak effects of CpG and Cryo, accounting for 26.09% (*P* = 0.012) and 18.83% (*P* = 0.029) of the total variation, respectively.

(g, h) Three-way ANOVA of fully activated CD137⁺ CTLs in treated and satellite tumors. CPI was the dominant factor contributing to variation in both compartments, accounting for 53.25% of the variation in treated tumors (*P* = 0.0001) and 52.99% in satellite tumors (*P* < 0.0001). In satellite tumors (h), Cryo and CpG also showed significant but smaller effects, explaining 14.52% (P = 0.0022) and 6.67% (P = 0.0251) of variation, respectively. No significant interaction effects or three-way interactions were observed in either compartment.

**Figure. 3**

(a, c, d, e) Comparison of early activated CTL(a), Th(c), early activated CD69^+^ CTL(d) and fully activated CD137^+^ CTL(e) frequencies between treated and satellite tumors within each group.

(b) CTL frequencies compared between pooled CPI-treated and non-CPI-treated cohorts.

(f) CD137+ CTL frequencies compared between pooled CPI-treated and non-CPI-treated cohorts.
(In all panels, ‘T’ indicates treated tumors and ‘S’ indicates satellite (untreated) tumors)

**Figure. 4**

(a, b) Three-way ANOVA revealed that in treated lesions, CPI and Cryo exerted significant main effects on effector CTL differentiation, accounting for 39.81% (*P* < 0.0001) and 17.67% (*P* = 0.0004) of the total variation, respectively. A modest but significant Cryo × CPI interaction (5.4% of variation, *P* = 0.0269) suggests potential synergy, further supported by the observed effector CTL frequency in the Cryo+CPI group (89.67%) exceeding the expected additive value of 87.13%. Although CpG alone did not reach significance, its contribution emerged through significant but modest interactions with CPI (8.18% of variation, *P* = 0.0085) and a three-way Cryo × CpG × CPI interaction (8.64%, *P* = 0.0071). The CpG+CPI group showed a slightly enhanced response compared to the expected additive value (83.73% vs. 83.4%), indicating a modest synergistic effect. In contrast, the triple combination group exhibited a lower-than-expected response (84.67% vs. 87.66%), suggesting that the addition of CpG to the Cryo+CPI regimen may dampen the synergy between Cryo and CPI, thereby limiting additive benefits. In satellite lesions, CPI and Cryo also showed significant main effects, contributing 55.65% (*P* < 0.0001) and 9.73% (*P* = 0.0297) of the total variation, respectively. CpG and all interaction terms were not statistically significant, indicating a reduced role for combinatorial effects in the distal tumor microenvironment.

(c, d) Effector Th frequencies were analyzed using three-way ANOVA. In treated lesions, only CPI showed a significant main effect, accounting for 37.12% of the total variation (*P* = 0.0037). Cryo and CpG did not show significant individual effects, and no significant two-way or three-way interactions were significant. In satellite lesions, none of the treatments reached statistical significance, though CPI approached the threshold (*P* = 0.052), contributing 15.54% of the total variation.

(e, f, g, h) Comparison of effector CTL(e), effector Th(f) frequencies, effector/naïve CTL ratio(g) and effector/naïve Th ratio(h) between treated and satellite tumors within each group.

(i) Effector CTL frequencies analyzed across groups between pooled CPI-treated vs. non-CPI-treated cohorts.

(j, k) Effector Th frequencies analyzed across groups (j) and pooled cohorts (k).
(In all panels, ‘T’ indicates treated tumors and ‘S’ indicates satellite (untreated) tumors)

**Figure. 5**

(a, b) Three-way ANOVA of PD-1 expression within CTLs across treatment groups. In treated tumors (a), both Cryo and CPI were major contributors to variation, accounting for 24.34% and 42.43% of the total variation, respectively (P < 0.0001 for both). Multiple significant interaction effects were observed, including Cryo × CPI (5.39%, P < 0.0001), Cryo × CpG (4.30%, P = 0.0002), CpG × CPI (2.41%, P = 0.0022), and a modest three-way interaction (17.48%, P < 0.0001). In satellite tumors (b), CPI remained the dominant contributor (49.29%, P < 0.0001), while Cryo and CpG accounted for 12.77% (P = 0.0014) and 18.53% (P = 0.0003) of the variation, respectively. Only the three-way interaction was statistically significant (4.03%, P = 0.0451), with no significant two-way interactions observed.

(c, d) Three-way ANOVA of CTLA-4 expression within CTLs across treatment groups. CPI was the dominant factor influencing CTLA-4 expression in CTLs, explaining 28.93% and 35.33% of total variation in treated and satellite tumors, respectively (P < 0.001). In treated tumors (c), Cryo also had a modest but significant main effect (7.72%, P = 0.0375), and a modest three-way interaction (10.21%, P = 0.019) was observed. In satellite tumors (d), a strong interaction between Cryo and CPI (30.06%, P < 0.0001) and a modest three-way interaction (14.62%, P = 0.0012) contributed to CTLA-4 variation. CpG had no significant main effect in either compartment.

(e, f, g) Comparison of PD-1 expression(e), CTLA-4 expression(f), and PD-1/CTLA-4 co-expression(g) between treated and satellite tumors within each group.

(h) PD-1 expression compared across monotherapies, Cryo-based dual therapies, and Triple treatment.

(i) PD-1/CTLA-4 co-expression on CTLs analyzed across groups.
(In all panels, ‘T’ indicates treated tumors and ‘S’ indicates satellite (untreated) tumors)

**Figure. 6**

(a, b) Three-way ANOVA revealed strong and consistent main effects of CpG and CPI on Treg frequencies in both treated and satellite tumors. In treated tumors, CpG and CPI accounted for 74.54% (*P* < 0.0001) and 9.36% (*P* = 0.0015) of the variation, respectively, with both treatments associated with reduced Treg frequencies. Cryo showed no significant main effect in treated lesions. In satellite tumors, all three treatments exhibited significant main effects: CpG (56.15%, *P* < 0.0001), CPI (16.76%, *P* < x`0.0001), and Cryo (7.41%, *P* = 0.0001). Multiple modest yet significant interaction effects were detected in satellite lesions, including a Cryo × CpG interaction (5.77%, *P* = 0.0004), a CpG × CPI interaction (6.75%, *P* = 0.0002), and a three-way interaction among Cryo, CpG, and CPI (1.42%, *P* = 0.0444).

(c) Comparison of Treg frequencies between treated and satellite tumors within each group.

(d) Treg frequencies compared between pooled CpG-treated and non-CpG-treated cohorts.
(In all panels, ‘T’ indicates treated tumors and ‘S’ indicates satellite (untreated) tumors)

**Figure 7. Unsupervised clustering and dimensionality reduction of CD45⁺ immune cells pooled across treatment groups.**

(a) PaCMAP projection of concatenated CD45⁺ cells from all treatment groups, where each group was first individually downsampled before pooling. Clustering was performed using X-shift, and each color denotes a distinct cluster.

(b) Same PaCMAP projection as in (a), with Cluster 13 cells highlighted in yellow and overlaid on the full dataset to visualize their distribution relative to other clusters.

(c) Bar plot showing the relative frequency of each cluster. Cluster 13 was the dominant population and was found to consist mainly of CD4⁻CD8⁻ cells.

**Figure 8. Dimensionality reduction and marker expression profiling of concatenated CD4⁺ and CD8⁺ T cells.**

(a–d) CD4⁺ and CD8⁺ T cells were pooled from all treatment groups, with 10,000 cells randomly downsampled per group and concatenated into a single file for unsupervised clustering. Clustering was performed using X-shift based on the expression of CD4, CD8, CD44, CD62L, CD69, CD137, PD-1, CTLA-4, FOXP3, and CD25. Clustered data were visualized using four dimensionality reduction methods: (a) t-SNE, (b) UMAP, (c) TriMAP, and (d) PaCMAP. Among the methods tested, PaCMAP provided the clearest separation of clusters with minimal overlap.

(e) Bar graph showing the relative frequency of each cluster among all concatenated CD4⁺ and CD8⁺ T cells.

(f) Heatmap of marker expression across the 16 identified clusters.

**Figure. 9**

(a, b) Three-way ANOVA revealed significant main effects of Cryo (27.48%, *P* = 0.0002) and CPI (22.16%, *P* = 0.0004) on MDSC frequencies in treated tumors, whereas CpG did not exhibit a significant main effect (*P* = 0.1666). In satellite tumors, all three treatments had significant main effects: CpG (40.9%, *P* < 0.0001), Cryo (24.77%, *P* = 0.0001), and CPI (5.10%, *P* = 0.0362), with CpG showing the strongest influence. A significant three-way interaction among Cryo, CpG, and CPI was observed in both treated (19.46%, *P* = 0.0008) and satellite tumors (8.04%, *P* = 0.0111). Notably, in both tumor sites, the observed MDSC frequencies in the Triple Therapy group (treated: 32.57%; satellite: 32.9%) were markedly higher than the expected additive values (0% in both), suggesting a strong antagonistic interaction that overrides the suppressive effects of CpG and CPI when combined with Cryo.

(c, d) Three-way ANOVA revealed significant main effects of CpG, CPI, and Cryo on M-MDSC frequencies in treated tumors, contributing 22.86% (*P* = 0.0035), 14.51% (*P* = 0.0149), and 11.06% (*P* = 0.03) of the variation, respectively. In satellite tumors, CpG again showed a dominant suppressive effect (56.36%, *P* < 0.0001), while Cryo also had a significant impact (13.13%, *P* = 0.0049); CPI, however, was not significant (*P* = 0.4524). A significant three-way interaction among Cryo, CpG, and CPI was detected in both treated (16.64%, *P* = 0.01) and satellite (6.968%, *P* = 0.0303) tumors. In both tumor sites, the observed M-MDSC frequencies in the Triple Therapy group (treated: 32.57%; satellite: 32.9%) were markedly higher than the expected additive values (treated: 0%; satellite: 1.4%), suggesting a strong antagonistic interaction whereby Cryo overrides the suppressive effects of CpG and CPI when all three treatments are combined.

(e, f) Three-way ANOVA revealed distinct regulatory effects of Cryo, CPI, and CpG on G-MDSC frequencies, with site-specific differences. In treated tumors, significant main effects were observed for Cryo (22.57%, *P* = 0.0015) and CPI (14.80%, *P* = 0.0069), whereas CpG had no significant effect (*P* = 0.43). Additionally, a strong Cryo × CpG interaction (20.35%, *P* = 0.0022) and a significant three-way interaction among Cryo, CpG, and CPI (11.07%, *P* = 0.0164) were detected. In satellite tumors, Cryo (22.32%, *P* = 0.0045) and CpG (20.52%, *P* = 0.006) showed significant main effects, while CPI did not (*P* = 0.1026). A significant Cryo × CpG interaction was also detected (10.05%, *P* = 0.0416).

(g, h, i) Comparison of MDSC(g), M-MDSC(h), and G-MDSC(i) frequencies between treated and satellite tumors within each group.

(j) Correlation study between PD-1 expression (PD1+ (% of CTL)) and MDSC frequency (CD11b+ Gr1+(% of CD45+ cells)).
(In all panels, ‘T’ indicates treated tumors and ‘S’ indicates satellite (untreated) tumors)

**Figure. 10**

(a, d) Frequencies of circulating effector CTLs (a) and effector Th cells (d) across treatment groups, defined as CD44⁺CD62L⁻ populations within CTLs or Ths, respectively.
(b, e) Correlations between effector T cell frequencies in peripheral blood and treated tumors for CTLs (b) and Th cells (e).
(c) Total Th frequencies (CD4⁺% of CD45⁺ cells) in peripheral blood.
(f, g, h) Frequencies of effector CTLs (f) and effector Th cells (g) compared between pooled CPI-treated and non-CPI-treated cohorts; Tregs (h) compared between pooled CpG-treated and non-CpG-treated cohorts. (**P* < 0.05)

**Figure. 11**

(a, b, c) CXCL1 (a), IL-2(b), and IFNγ (c) levels measured on Day 0, 2, and 7 across groups.

(d, e) Correlation analyses between Day 2 cytokine levels and intratumoral Treg frequencies.

(f, g) Day 2 IL-10 (f) and TNF-α (g) levels compared between CpG-treated and non–CpG-treated cohorts.

(h) Day 7 IL-6 levels compared between Cryo-treated and non–Cryo-treated cohorts.

(i) Quantification of CD8⁺ T cells per 20× HPF in the regions of treated and satellite liver.

(g) Representative IHC images showing CD8⁺ T cells (brown) in the liver for Control, Cryo, and Triple therapy groups. (**P* < 0.05)
